# Excess mortality associated with the COVID-19 pandemic in Latvia: a population-level analysis of all-cause and noncommunicable disease deaths in 2020

**DOI:** 10.1186/s12889-022-13491-4

**Published:** 2022-06-03

**Authors:** Inese Gobiņa, Andris Avotiņš, Una Kojalo, Ieva Strēle, Santa Pildava, Anita Villeruša, Ģirts Briģis

**Affiliations:** 1 Institute of Public Health, Riga Stradiņš University, Anninmuizas Boulevard 26a, Riga, LV-1067 Latvia; 2 Faculty of Biology and Faculty of Geography and Earth Sciences, University of Latvia, Jelgavas street 1, Riga, LV-1004 Latvia; 3 Centre for Disease Prevention and Control, Duntes 22, k-5, Riga, LV-1005 Latvia; 4 Department of Public Health and Epidemiology, Riga Stradiņš University, Kronvalda Boulevard 9, Riga, LV-1010 Latvia

**Keywords:** Mortality, COVID-19, Excess deaths, Noncommunicable diseases

## Abstract

**Background:**

Age-standardised noncommunicable disease (NCD) mortality and the proportion of the elderly population in Latvia are high, while public health and health care systems are underresourced. The emerging COVID-19 pandemic raised concerns about its detrimental impact on all-cause and noncommunicable disease mortality in Latvia. We estimated the timing and number of excess all-cause and cause-specific deaths in 2020 in Latvia due to COVID-19 and selected noncommunicable diseases.

**Methods:**

A time series analysis of all-cause and cause-specific weekly mortality from COVID-19, circulatory diseases, malignant neoplasms, diabetes mellitus, and chronic lower respiratory diseases from the National Causes of Death Database from 2015 to 2020 was used by applying generalised additive modelling (GAM) and joinpoint regression analysis.

**Results:**

Between weeks 14 and 52 (from 1 April to 29 December) of 2020, a total of 3111 excess deaths (95% PI 1339 – 4832) were estimated in Latvia, resulting in 163.77 excess deaths per 100 000. Since September 30, with the outbreak of the second COVID-19 wave, 55% of all excess deaths have occurred. Altogether, COVID-19-related deaths accounted for only 28% of the estimated all-cause excess deaths. A significant increase in excess mortality was estimated for circulatory diseases (68.91 excess deaths per 100 000). Ischemic heart disease and cerebrovascular disease were listed as the underlying cause in almost 60% of COVID-19-contributing deaths.

**Conclusions:**

All-cause mortality and mortality from circulatory diseases significantly increased in Latvia during the first pandemic year. All-cause excess mortality substantially exceeded reported COVID-19-related deaths, implying COVID-19-related mortality during was significantly underestimated. Increasing mortality from circulatory diseases suggests a negative cumulative effect of COVID-19 exposure and reduced access to healthcare services for NCD patients.

**Supplementary Information:**

The online version contains supplementary material available at 10.1186/s12889-022-13491-4.

## Background

Mortality surveillance is crucial for real-time monitoring of COVID-19 deaths to follow the dynamics of SARS-CoV-2 epidemics and the impact of public health measures. However, COVID-19 mortality data limit our understanding of the true burden of the COVID-19 pandemic [1, 2]. Therefore, excess all-cause mortality is widely used to estimate the total impact of the COVID-19 pandemic, as it includes not only COVID-19 confirmed or related deaths but also mortality from other causes attributable to pandemic conditions [3–5].

Existing evidence suggests that certain noncommunicable diseases (NCD) result in higher mortality and more severe cases of COVID-19 [6–8]. However, the COVID-19 pandemic has resulted in disruptions in healthcare services that have affected the supply and demand of NCD care [9–11].

Latvia is one of the countries at high risk of both the proportion of the population aged 70 years and above and the rate of years lived with disability (YLD) [12], and it has one of the highest age-standardised NCD mortality rates in Europe [13]. Moreover, Latvia has one of the lowest gross domestic product (GDP) shares to health care in the European Union (EU) [14].

Latvia experienced two COVID-19 waves in 2020. During the first wave of the COVID-19 pandemic in spring 2020, 14-day COVID-19 case and death rates in Latvia were among the lowest in EU countries [15]. The first emergency in Latvia was declared from 13 March until 9 June 2020 [16], and substantial restrictions on the provision of planned inpatient and outpatient health care services by the order of the Minister of Health occurred between 27 March and 9 June 2020 [17]. In October 2020, a more severe COVID-19 second wave emerged [15], but formal restrictions on planned health care services were not reimposed.

During the first COVID-19 wave in Latvia, a substantial decrease in the total number of consultations for patients with NCD provided by both general practitioners and specialists was observed [18]. Altogether, the ageing population, high prevalence of NCD, and underresourced healthcare system combined with disruptions in health care services in Latvia during the COVID-19 pandemic's first year have raised concerns about adverse effects on patients with NCD.

We aimed to investigate the all-cause mortality and cause specific trends and excess deaths from selected NCD by using the National Database of Causes of Death. We estimated (1) the timing and the number of all-cause excess deaths after accounting for population size, temporal trends, and seasonal variations; 2) the excess deaths related to COVID-19 (caused and contributing mortality); and 3) cause-specific excess deaths from cardiovascular diseases, malignant neoplasms, diabetes mellitus, and chronic lower respiratory diseases as those accounting for the majority of mortality in Latvia [19].

## Methods

The study was implemented within the National Research Program VPP-COVID-2020/1-0011 initiative to determine the impact of COVID-19 on health care and public health in Latvia by following the study protocol reviewed and approved by the Committee of Ethics of Riga Stradiņš University (2-PĒK-4/36/2022).

To conduct a time series analysis of weekly mortality in Latvia, all daily deaths from 2015 to 2020 were requested and extracted from the Causes of Death Database with the Centre for Disease Prevention and Control (CDPC) permission in Latvia. In addition, to estimate COVID-19-related mortality, all deaths with COVID-19 recorded as an underlying (COVID-19 caused) or contributing cause (COVID-19 contributing) were extracted. The ratio of all recorded COVID-19-related deaths to the overall number of excess deaths was used to compute the fraction of excess mortality attributed to COVID-related deaths with Wilson’s 95% confidence intervals of binomial distribution. In cases of division by zero, the imputation of zero was employed.

All-cause and cause-specific mortality from the following disease groups were studied using ICD-10 codes: (1) COVID-19 (U07.1 – U07.2), (2) circulatory diseases (hypertensive diseases (I10 – I16), ischemic heart diseases (I20 – I25) and cerebrovascular diseases (I60 – I69), (3) malignant neoplasms (C00 – C97), (4) diabetes mellitus (E10 – E14), and (5) chronic lower respiratory diseases (J40–J47). All data were accessed on 27 February 2021.

For the analysis, the baseline population size estimates for each month were obtained from the Central Statistical Bureau of Latvia, which records the monthly population at the start of the first week. The weekly national population size was interpolated with a linear regression between the death rates of the first weeks of the months. A regression coefficient was used to calculate population counts in the following weeks.

Generalised additive modelling (GAM) was applied to estimate the timing and the amount of weekly excess mortality in 2020 by fitting two separate models for all-cause and cause-specific mortality^1^. GAMs were implemented with the software R package ‘mgcv’ [20]. The principal model is defined as log(*E*(*y*_*i*_)) = *α* + *β* × *Year*_*i*_ + *f*(*WOY*_*i*_) + *o*(log(*Population*_*i*_)), *y*_*i*_~*Poisson*(*λ*), corresponding to the main effects model. The year of death was used as a factor (“ *Year*_*i*_ ”) accounting for nonlinear seasonal changes in mortality with a smoothing component (“ *f* ”) over the week of the year (“ *WOY*_*i*_ ”). The first week of each year began on 1 January, resulting in 52 full (seven-day) weeks, ensuring equal time intervals while losing only one to two days of data per year. Additionally, both fixed (corresponding to a knot per week) and machine-selected numbers of functions were compared for describing seasonality. The following basis functions with smoothing components were evaluated: thin-plate splines, cubic regression splines, and cyclic cubic splines. Weekly sums of death cases (“ *y*_*i*_ ”) were used as a Poisson distributed response with a logarithmic link function. To account for Latvia's declining population, an offset component with a natural logarithmically transformed weekly population size ("o(log(Population i))") was used.

Before the final analysis, the effects of different baseline lengths on the relative risk of all-cause mortality in 2020 were evaluated. Four different timelines for estimating expected deaths in 2020, each beginning with the first week of 2015 and ending with meaningful events in 2020, were compared: (1) date of the first registered COVID-19 case (2 March 2020); (2) date of the first state emergency (12 March 2020); (3) date when substantial restrictions of planned health services were initiated (27 March 2020); and (4) date of the first registered COVID-19 death (3 April 2020). To select a baseline period for final estimates, the relative risks and the smoothing function by its effective degrees of freedom (edf) were compared. Overall, 24 models for baseline length selection were evaluated: 4 baseline lengths * 3 basis functions * 2-knot types (fixed to k=52 or not). All the models produced a considerably similar effect in 2020 (relative risks) and seasonality. This study aimed to investigate the effect on mortality; thus, a period between 2015 and the first confirmed death case in 2020 as the baseline was chosen. The second-order Akaike’s information criterion (AICc) value in the final analysis was used to select the best generalising model [21]. Additional file 1 contains the year effects from the best model as relative risks with 95% confidence intervals and a description of the basis functions and model fit statistics.

**Fig. 1:**
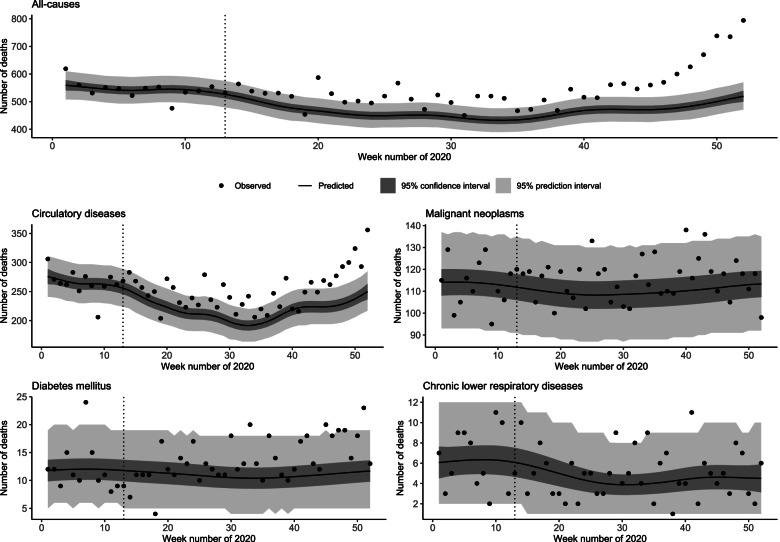
All-cause and cause-specific mortality groups with the observed and predicted number of deaths in Latvia, 2020

Excess deaths for all-cause and selected cause-specific mortality were estimated by subtracting the model-predicted deaths from observed weekly deaths from 1 April 2020 (week 14) to 29 December 2020 (week 52), with 95% prediction intervals to establish the lower and upper bounds of the estimate. The total number of excess deaths during the studied period was calculated by summing the number of excess deaths each week. The observed and model-predicted absolute numbers of deaths with 95% intervals for the confidence of the mean and the posterior distribution were used to visualise the timing and amount of excess mortality in 2020 (Fig. 1). Weeks with a statistically significant excess mortality were defined as those with excess mortality above the upper bound of the 95% PI. Data processing and visualisation were performed in the ‘tidyverse’ ecosystem [22].

In addition, joinpoint regression analysis (Joinpoint Regression Program; version 4.7.0.0, February 2019, US National Cancer Institute) was used to determine trends in mortality from 2015 to 2020. A Monte Carlo permutation method with 4499 replicates was used for significance tests. The smallest number of joinpoints was initially chosen and then increased to five by determining the statistical significance. Additional file 2 contains figures depicting the joinpoint regression results.

The weekly percent change (WPC) in rates between trend-change points and the average weekly percent change (AWPC) with the corresponding 95% confidence intervals (CIs) were estimated. Parallelism and coincidence tests were used to determine whether two regression functions were identical or parallel [23]. Statistical significance for two-sided P values was set to <0.05.

## Results

### All-cause excess mortality

In 2020, the total mortality in Latvia was expected to be lower than that in the previous five years (Additional file 1). With 21291 total deaths between weeks 14 (1 April) and 52 (29 December) in 2020, 3111 (95% PI 1339 – 4832) excess deaths were estimated, resulting in 163.77 excess deaths per 100 000 (95% PI 70.49– 254.37) Table 1.

**Table 1:** Excess and COVID-19-related deaths in Latvia from weeks 14 to 52 in 2020

WOY	Dates	Observed deaths	Expected deaths (95%PI)	Estimated excess deaths (95%PI)	Estimated excess mortality per 100 000 (95%PI)	COVID-19 caused deaths	COVID-19 contributing deaths	Total COVID-19 related deaths	Excess deaths (%) related to COVID-19 (95%CI)
14	01/04 – 07/04	564	518 (472 to 565)	46 (-1 to 92)	2.42 (-.0.05 to 4.83)	2	0	2	4.35 (1.20 to 14.53)
15	08/04 – 14/04	538	507 (462 to 554)	31 (-16to 76)	1.63 (-.0.84 to 3.99)	2	1	3	9.68 (3.35 to 24.90)
16	15/04 – 21/04	530	495 (450 to 542)	35 (-12 to 80)	1.84 (-0.63 to 4.2)	2	1	3	8.57 (2.96 to 22.38)
17	22/04 – 28/04	531	485 (439 to 531)	46 (0 to 92)	2.42 (0 to 4.83)	2	2	4	8.70 (3.43 to 20.32)
18	29/04 – 05/05	519	477 (431 to 524)	42 (-5 to 88)	2.21 (-.0.26 to 4.62)	2	1	3	7.14 (2.46 to 19.01)
19	06/05 – 12/05	454	471 (428 to 516)	-17 (-62 to 26)	-0.89 (-3.26 to 1.37)	0	0	0	0
20	13/05 – 19/05	587	466 (422 to 512)	121 (75 to 165)	6.36 (3.94 to 8.67)	1	1	2	1.65 (0.45 to 5.83)
21	20/05 – 26/05	529	461 (417 to 506)	68 (23 to 112)	3.57 (1.21 to 5.89)	1	1	2	2.94 (0.81 to 10.10)
22	27/05 – 02/06	498	456 (413 to 500)	42 (-2 to 85)	2.21 (-0.11to 4.47)	0	1	1	2.38 (0.12 to 12.32)
23	03/06 – 09/06	502	451 (408 to 496)	51 (6 to 94)	2.68 (0.32 to 4.94)	1	0	1	1.96 (0.10 to 10.30)
24	10/06 – 16/06	495	449 (405 to 494)	46 (1 to 90)	2.42 (0.05 to 4.73)	1	2	3	6.52 (2.24 to 17.50)
25	17/06 – 23/06	520	449 (407 to 494)	71 (26 to 113)	3.73 (1.37 to 5.94)	0	0	0	0 (0 to 5.13)
26	24/06 – 30/06	567	450 (407 to 494)	117 (73 to 160)	6.16 (3.84 to 8.42)	0	0	0	0 (0 to 3.18)
27	01/07 – 07/07	509	451 (408 to 496)	58 (13 to 101)	3.05 (0.68 to 5.31)	0	0	0	0 (0 to 6.21)
28	08/07 – 14/07	472	451 (408 to 497)	21 (-25 to 64)	1.10 (-1.32 to 3.37)	0	1	1	4.76 (0.24 to 22.67)
29	15/07 – 21/07	524	449 (407 to 494)	75 (30 to 117)	3.95 (1.58 to 6.16)	0	0	0	0 (0 to 4.87)
30	22/07 – 28/07	497	445 (402 to 488)	52 (9 to 95)	2.74 (0.47 to 5)	0	0	0	0 (0 to 6.88)
31	29/07 – 04/08	450	440 (397 to 484)	10 (-34 to 53)	0.53 (-1.79 to 2.79)	1	0	1	10.00 (0.51 to 40.42)
32	05/08 – 11/08	520	435 (393 to 479)	85 (41 to 127)	4.47 (2.16 to 6.68)	0	0	0	0 (0 to 4.32)
33	12/08 – 18/08	520	433 (390 to 476)	87 (44 to 130)	4.58 (2.32 to 6.84)	0	1	1	1.15 (0.06 to 6.23)
34	19/08 – 25/08	512	432 (390 to 476)	80 (36 to 122)	4.21 (1.9 to 6.42)	0	0	0	0 (0 to 5.92)
35	26/08 – 01/09	467	434 (391 to 478)	33 (-11 to 76)	1.74 (-0.58 to 4)	1	0	1	3.03 (0.16 to 15.32)
36	02/09 – 08/09	472	438 (395 to 481)	34 (-9 to 77)	1.79 (-0.47 to 4.05)	1	0	1	2.94 (0.15 to 14.92)
37	09/09 – 15/09	506	445 (402 to 488)	61 (18 to 104)	3.21 (0.95 to 5.48)	0	0	0	0 (0 to 5.92)
38	16/09 – 22/09	468	453 (410 to 498)	15 (-30 to 58)	0.79 (-1.58 to 3.05)	1	0	1	6.67 (0.34 to 29.82)
39	23/09 – 29/09	545	462 (417 to 507)	83 (38 to 128)	4.37 (2 to 6.74)	0	1	1	1.20 (0.06 to 6.51)
40	30/09 – 06/10	516	468 (423 to 514)	48 (2 to 93)	2.53 (0.11 to 4.9)	2	0	2	4.17 (1.15 to 13.98)
41	07/10 – 13/10	514	471 (426 to 517)	43 (-3 to 88)	2.27 (0 to 4.64)	1	0	1	2.33 (0.12 to 12.06)
42	14/10 – 20/10	561	472 (428 to 517)	89 (44 to 133)	4.69 (2.32 to 7.01)	6	0	6	6.74 (3.13 to 13.94)
43	21/10 – 27/10	565	471 (427 to 517)	94 (48 to 138)	4.95 (2.53 to 7.27)	9	6	15	15.96 (9.92 to 24.67)
44	28/10 – 03/11	546	471 (427 to 516)	75 (30 to 119)	3.95 (1.58 to 6.27)	13	8	21	28.00 (19.10 to 39.04)
45	04/11 – 10/11	560	471 (426 to 516)	89 (44 to 134)	4.69 (2.32 to 7.06)	9	6	15	16.85 (10.49 to 25.96)
46	11/11 – 17/11	570	473 (430 to 518)	97 (52 to 140)	5.11 (2.74 to 7.38)	28	11	39	40.21 (31.00 to 50.16)
47	18/11 – 24/11	600	477 (433 to 523)	123 (77 to 167)	6.49 (4.06 to 8.81)	40	12	52	42.28 (33.91 to 51.11)
48	25/11 – 01/12	626	483 (436 to 530)	143 (96 to 190)	7.54 (5.06 to 10.02)	48	20	68	47.55 (39.54 to 55.69)
49	02/12 – 08/12	670	491 (444 to 537)	179 (133 to 226)	9.44 (7.02 to 11.92)	79	28	107	59.78 (52.46 to 66.68)
50	09/12 – 15/12	738	500 (454 to 549)	238 (189 to 284)	12.56 (9.97 to 14.98)	112	41	153	64.29 (58.02 to 70.10)
51	16/12 –22/12	735	510 (463 to 558)	225 (177 to 272)	11.87 (9.34 to 14.35)	139	35	174	77.33 (71.43 to 82.32)
52	23/12 – 29/12	794	519 (471 to 570)	275 (224 to 323)	14.51 (11.82 to 17.05)	158	42	200	72.73 (67.18 to 77.65)
TOTAL	01/04 – 29/12	21291	18180	3111 (1339 to 4832)	163.77 (70.49 to 254.37)	662	222	884	28.42 (26.86 to 30.03)

Over the study period of 1 April to 29 December 2020, there was a statistically significant increase in excess mortality for 26 weeks. Following week 40, a total of 1718 (95% PI 1114 – 2214) excess deaths were estimated, accounting for 55% of the total excess deaths in 2020 (Fig. 1).

Joinpoint analysis also showed that the observed all-cause mortality patterns in 2020 (AWPC= 0.62; 95% CI 0.38–0.86) were significantly different from those in the previous five years (AWPC= -0.15; 95% CI -0.42–0.13). Overall, all-cause mortality decreased until week 40 of 2020 but then increased by 3.72% (95% CI 2.82. – 4.63) per week, whereas in 2015 – 2019, the autumn-winter increase was fourfold lower and began at week 34 Table 2.

**Table 2:** All-cause and NCD mortality trends in Latvia in 2020 compared to the average from 2015 to 2019: Joinpoint regression analysis

Years	# Joinpoints	Joinpoint week (95% CI)	WPC^a^ (95% CI)	AWPC^b^ (95% CI)
All-cause mortality				
Test for coincidence: *p*<0.001; Test for parallelism: *p*<0.001				
			-0.22 (-0.64–0.19)	
		13 (7–16)		
2015–2019			-2.65* (-4.88– -0.37)	-0.15 (-0.42–0.13)
	3	18 (14–26)		
			-0.46* (-0.79– -0.14)	
		34 (30–39)		
			0.90* (0.65– 1.15)	
			-0.32* (-0.48– -0.15)	
2020	1	40 (36–43)		0.62* (0.38–0.86)
			3.72* (2.82–4.63)	
Circulatory diseases (I10–I16; I20–I25; I60–I69)				
Test for coincidence: *p*<0.001; Test for parallelism: *p*<0.001				
			-0.31 (-1.14–0.53)	
2015–2019	2	10 (5–17)		
			-1.43* (-1.68–-1.17)	-0.17 (-0.38–0.03)
		32 (29–34)		
			1.28* (1.00–1.57)	
			-0.50* (-0.72–0.27)	
2020	1	40 (33–43)		0.40* (0.06–0.74)
			3.36* (2.04–4.69)	
Malignant neoplasms (C00–C97)				
Test for coincidence: *p*<0.05				
2015–2019			-0.33* (-0.57–0.09)	
	1	25 (16–39)		-0.03 (-0.19–0.12)
			0.23* (0.03–0.43)	
2020	0		0.08 (-0.08–0.24)	0.08 (-0.08–0.24)
Test for parallelism: *p*=0.55				
			-0.28* (-0.51– -0.06)	
2015–2019 and 2020 combined	1	24 (15–35)		-0.02 (-0.16–0.12)
			0.20* (0.03–0.37)	
Diabetes (E10–E14)				
Test for coincidence: *p*<0.001; Test for parallelism: *p*<0.001				
2015–2019	0		-0.30* (-0.59– -0.01)	-0.30*(-0.59– -0.01)
2020	0		0.83* (0.34–1.32)	0.83* (0.34–1.32)
Chronic lower respiratory diseases (J40–J47)				
Test for coincidence: *p*=0.465; Test for parallelism: p=0.381				
			-1.85* (-2.63– -1.06)	
2015–2019 and 2020 combined	1	29 (23–38)		-0.70* (-1.36– -0.03)
			0.73 (-0.41– 1.88)	

^a^ WPC – weekly percent change; ^b^ AWPC – average weekly percent change

### Excess mortality related to COVID-19

The first two COVID-19 deaths were reported on week 14 Table 1. In Latvia, the registered COVID-19-caused mortality was 34.85 per 100 000 (*n* = 662) in 2020. The SARS-CoV-2 virus was not laboratory-confirmed in only three out of a total of 662 COVID-19-caused deaths (coded as U07.2), while all the COVID-19 contributing registered deaths (*n* = 222) were laboratory confirmed (coded as U07.1.). Most COVID-19-contributing deaths (58.6%) were deaths from cardiovascular diseases (I00-I99). Malignant neoplasms (C00-C97) accounted for 19.8% of COVID-19 contributing deaths, whereas diabetes mellitus (E10–E14) accounted for 4.5%, but 17.1% of COVID-19-contributing deaths were attributed to diseases other than those studied. COVID-19 was not identified as a contributing cause of death for chronic lower respiratory deaths.

Overall, 96.4% of all COVID-19-related deaths occurred between weeks 40 and 52 Table 1. Additionally, between weeks 40 and 52, when the most significant increase in excess deaths occurred, COVID-19-related deaths contributed 49.65% (95% CI 47.29 – 52.02) of the total estimated all-cause excess deaths. Altogether, COVID-19-related deaths accounted for 28.42% (95% CI 26.86 – 30.03) of the estimated all-cause excess deaths in total.

### Excess mortality and mortality trends of noncommunicable diseases

#### Circulatory diseases

The expected mortality from circulatory diseases in 2020 was lower than that in the preceding five years (Additional file 1). In 2020, 1309 (95% PI 88 – 2476) excess deaths from circulatory diseases were estimated. There was a significant increase in excess mortality from circulatory diseases beginning at week 20 for a total of 18 weeks during the studied period. However, a more rapid and consistent increase occurred after week 40. Overall, 68.91 excess deaths per 100 000 (95% PI 4.63 – 130.35) due to circulatory diseases in 2020 were estimated (Fig. 1).

Joinpoint analysis also revealed a different trend in circulatory disease mortality in 2020 compared to the previous five-year average (*p*<0.001; Table 2). By week 40 of 2020, mortality from circulatory disorders had declined, but then the increase was followed that more than twice as large compared to the autumn-winter increase in mortality from circulatory diseases in the preceding five years.

#### Malignant neoplasms

The estimated excess deaths from malignant neoplasms in 2020 were 208 (95% PI -656 – 1025). During the studied period, three weeks had a significant positive excess of death, but the total excess mortality from malignant neoplasms was not significant (10.94 per 100 000; 95% PI -34.53 – 53.96; Fig. 1).

While the joinpoint regression mean functions for 2020 and 2015 to 2019 were different (*p*<0.05), the malignant neoplasm mortality trends were parallel (*p*=0.55), and both showed no significant changes from 2015 to 2020 Table 2.

#### Diabetes mellitus

Three weeks between weeks 14 and 52 in 2020 were found to have a significant positive excess mortality from diabetes mellitus, whereas one week (week 18) had a significant negative excess mortality (Fig. 1). Overall, the excess mortality from diabetes mellitus with an estimated 113 (95% PI -78 – 353) excess deaths, resulting in excess mortality of 5.95 per 100 000 (95% PI --9.37 – 18.58), was not significant.

Diabetes mellitus mortality trends differed significantly between 2015–2019 and 2020 (*p*<0.001). On average, mortality from diabetes mellitus decreased in the period from 2015 to 2019, with no further significant changes in the trend observed throughout the year Table 2. A slight discrepancy between the GAM and the joinpoint analysis results occurs due to the GAM evaluating the effect of individual years versus pooled baseline data in joinpoint analysis. However, both methods agree that the observed mortality from diabetes mellitus in 2020 was higher than expected. Thus, we regard these findings as additional overall support rather than an artefact.

#### Chronic lower respiratory diseases

The model predicted a statistically significant increase in mortality from chronic lower respiratory diseases from 2015 to 2018, but no significant changes were expected in 2019 and 2020 (Additional file 1). In total, 14 (95% PI -174 – 154) excess deaths from chronic lower respiratory diseases were estimated, resulting in a 0.74 per 100 000 (95% PI -9.16 – 8.11) excess mortality that was not significant. In 2020, two weeks were identified as having a significant excess of deaths from chronic lower respiratory diseases, and observed mortality was distributed around the predicted mean (Fig. 1).

Between 2015 and 2020, joinpoint regression analysis revealed no statistically significant differences in the trends in mortality from chronic lower respiratory diseases in Latvia Table 2.

## Discussion

### All-cause excess mortality

Excess mortality varies significantly across countries [24, 25]. While excess mortality has fewer cross-country comparability constraints than COVID-19 mortality, excess mortality results are affected by the methods and baselines used for the analysis. Islam et al. estimated fewer excess deaths (n = 820, 95% CI 490 – 1100) for Latvia in 2020 by using a unified approach for excess mortality estimates for high-income countries by fitting aggregate mortality data from 2016 to 2019 from the Human Mortality Database (HMD) [25]. The analysis employed incomplete data on COVID-19 deaths in Latvia.

In this study, mortality data between 2015 and 2020 using the National Database of Causes of Death were analysed, allowing the study of cause-specific NCD deaths and distinguishing between recorded COVID-19-caused and contributing deaths for the NCD investigated. We aimed to increase the reliability of our estimates by utilising complete mortality data at the country level and minimising potential errors when modelling cause-specific excess deaths from NCD and calculating COVID-19-related deaths as a result of reporting and registration delays. Differences in underlying death rates may impact comparisons of excess mortality data across countries. As Latvia has one of the highest rates of noncommunicable disease mortality among EU countries because it has underresourced public health and health care systems [14], the relatively lower rates of excess mortality during the first COVID-19 wave may be explained by timely and stringent national public health measures in spring 2020 [15]. However, excess mortality is also affected by the timing and dynamics of COVID-19. In 2020, Latvia's two COVID-19 waves were markedly different, with the second COVID-19 outbreak accounting for more than 90% of total COVID-19-related deaths in the first pandemic’s year. Comparing the number of excess deaths between the first and complete second COVID-19 outbreaks continued in 2021 requires additional research. However, our study found a significant all-cause excess mortality between the two COVID-19 waves in 2020, when reported COVID-19 cases and deaths were low, indicating the pandemic continued impact throughout the first year.

### Excess mortality due to COVID-19

In 2020, excess deaths were significantly greater than reported COVID-19 deaths in the majority of countries [25]. This implies that estimating the pandemic's effects solely based on COVID-19 deaths significantly underestimates the true burden of the pandemic and associated policy measures or behaviour changes. Our study found a large discrepancy between reported COVID-19 deaths and excess mortality, indicating underreporting of COVID-19 deaths that may be caused by several factors, including insufficient testing, significant reductions in healthcare services, delayed care, and misclassification of COVID-19 deaths.

In Latvia, COVID-19 death rates remained low during the first wave of the pandemic. Between June and September 2020, the number of tests performed per 100 000 persons remained relatively constant in Latvia [15]. By October 2020, the rate of weekly COVID-19 testing in Latvia had significantly increased. In November 2020, when the second state of emergency was declared [26], health care and long-term care institutions began routinely performing massive COVID-19 testing. Additionally, COVID-19 testing for the public became accessible without general practitioners’ referral. According to our findings, the gap between COVID-19 deaths and excess deaths narrowed significantly after week 40. The gap between excess mortality and COVID-19 confirmed mortality in 2020 is associated with the COVID-19 testing rate [27]. However, the ratio between excess deaths and confirmed COVID-19 mortality is influenced not only by the number of tests, but also by the testing bias that results from the variations in testing strategies and the tested subpopulations during different time periods [28].

Furthermore, underdiagnosis of COVID-19 may occur due to a substantial reduction in healthcare services. A systematic review suggests that healthcare utilisation declined by approximately one-third during the pandemic [29], which may be due to overstretched health systems and healthcare avoidance [30, 31]. Fear of COVID-19 exposure and suspension of planned healthcare services during the first COVID-19 wave in Latvia may negatively affect NCD health care and increase the population's avoidance of seeking medical care, contributing to underestimating the COVID-19 cases, although this requires further research.

COVID-19 has presented significant challenges to healthcare institutions and practitioners due to unprecedented uncertainty [32]. Our previous study suggested that during the first COVID-19 wave in Latvia, clinicians experienced a sense of confusion, and fear of rapid change, as a result of the unknown disease [18]. Facing a new disease may impact the accuracy and reliability of COVID-19 death records, especially early in the pandemic, when the case definition was not initially clear and testing was limited [33]. During the first COVID-19 wave, a significant excess of pneumonia and influenza deaths were found in the USA, suggesting that COVID-19 deaths may have been misclassified [34, 35]. In addition, comorbidities may complicate the assignment of COVID-19 and other illnesses to either underlying or contributing causes of death on the death certificate. Thus, the differentiation between COVID-19-caused deaths, deaths contributed by COVID-19, and deaths from other causes in people with positive COVID-19 tests adds to the already existing variability in causes of death certification, thereby hampering the international comparability of COVID-19 mortality [36].

### Excess mortality due to noncommunicable diseases

Cardiovascular disease (CVD) is one of the most prevalent underlying conditions associated with increased mortality from COVID-19 [6]. Evidence shows that COVID-19 may either cause new cardiac pathologies or exacerbate preexisting cardiovascular diseases [37]. We found statistically significant excess mortality from circulatory diseases in Latvia during the first COVID-19 pandemic year. COVID-19 mitigation strategies and overstretching the health system may negatively affect disease management and care for CVD patients. Several countries have reported a decrease in hospital admissions for acute coronary syndromes during the COVID-19 pandemic [38, 39] and a significant decline in primary percutaneous coronary intervention procedures [40]. The National Health Service data analysis suggests a considerable reduction in the supply and demand for cardiovascular disease inpatient and outpatient public services in Latvia during the first COVID-19 emergency state [18]. Some studies found an increase in out-of-hospital CVD mortality during the COVID-19 pandemic [41, 42], which requires additional investigation in Latvia.

Cancer patients are vulnerable to increased risks of contracting and dying from SARS-CoV-2 infection [43]. COVID-19 and cancer may also interact, resulting in misclassification of the underlying cause of death [44]. In 2020, excess mortality from malignant neoplasms was not significant. In Latvia, cancer care services were maintained during the first COVID-19 wave to ensure treatment continuity [17]. However, disruptions in organised cancer screening programmes and oncological surgeries occurred as a result of those services being suspended in different periods from March to April 2020. Thus, the impact of the COVID-19 pandemic on cancer care services and mortality trends of malignant neoplasms should be monitored, as the pandemic may affect more cancer patients, particularly those with comorbidities, in the long run [45].

Diabetes patients have been considered a high-risk group since the start of the COVID-19 epidemic [46]. During the first pandemic year, more studies show an increase in mortality from diabetes [5, 47, 48]. Overall, we found that the observed mortality from diabetes mellitus in 2020 was distributed above the predicted mean. Diabetes mellitus was recorded in only 4.5% of COVID-19 contributing deaths. We did not study the proportion of COVID-19-related deaths in people with prior diabetes. The reporting of diabetes on death certificates is highly variable, and diabetes as the contributing cause is underreported [49].

The excess mortality from chronic lower respiratory diseases was low and not significant in Latvia during the first COVID-19 pandemic year. While it appears reasonable to assume that patients with chronic respiratory illnesses are at increased risk of contracting COVID-19 infection and suffering adverse outcomes, existing data are inconsistent. The umbrella review on comorbidities and the outcomes of COVID-19 shows that preexisting chronic obstructive respiratory disease (COPD) increases the risk of hospitalisation, whereas bronchial asthma does not [46]. Reduced transmission of other respiratory viruses and outdoor air pollutants or improved COPD self-management due to an increase in bronchial asthma medication prescriptions prior to the lockdown might explain the decrease in the exacerbations of chronic respiratory diseases [50, 51].

Given the similarity of the symptoms, concerns about recognising and differentiating COVID-19 from COPD remain [52]. In our study, COVID-19 was not listed as a contributing cause of death in any of the chronic lower respiratory deaths. While we cannot prove the validity or sequence of cause-of-death chains, some of our preliminary findings indicate the critical importance of conducting additional research on COVID-19 deaths and associated comorbidities.

The COVID-19 pandemic may increase noncommunicable disease mortality in several ways. Social distancing may negatively affect noncommunicable disease-related health behaviours and increase isolation [53–55]. Overall, fear of contracting COVID-19 and a sense of vulnerability appear to discourage patients from seeking help and delay care seeking [56]. However, one of the major concerns during the COVID-19 pandemic is ensuring the continuity of care for disease control and management in patients with NCD. The COVID-19 pandemic resulted in significant reductions in the demand for and supply of health services that may have led to missing care and increased mortality from NCD [39, 57–61]. Our previous analysis of National Health Services data showed a significant reduction in the supply and demand for NCD inpatient and outpatient public health care services in Latvia during the first COVID-19 emergency state [18].

Although further research into the individual-level associations between health care service utilisation and NCD mortality is necessary to provide definite and conclusive evidence, our findings suggest that suspending planned health services during the first wave of COVID-19 and overburdening the healthcare system during the second COVID-19 outbreak may have had a negative impact on NCD mortality during the first year of the pandemic.

### Limitations

By modelling the expected mortality for 2020, we accounted for nonlinear seasonal trends, changes in population size and year-specific mortality rates; however, we did not standardise for age and sex, or COVID-19 testing rates. However, the changes in the population structure between 2015 and 2020 are considered minor [62]. A previous study by Islam et al. showed that in Latvia, the greatest proportion of all-cause excess deaths are attributed to those aged 65 or older [25]. When estimating the age-specific risk of death during the COVID-19 pandemic in a population aged 70 or more stratified by level of care, a study in Sweden suggested that health status and comorbidities may play a more important role in COVID-19-associated deaths than age itself [4].

We recognise the need to study all-cause and cause-specific mortality in more detail to improve our understanding of the pandemic impact of COVID-19, which may reveal different patterns in distinct population subgroups. We did not consider COVID-19 testing rates in our analysis. Thus, our study could not quantify the impact of testing on the underreporting of COVID-19 related deaths.

This study was limited to those NCD causing the most deaths in Latvia. Nonetheless, the COVID-19 pandemic has also an adverse effect on other NCD, i.e., neurological and nephrological diseases [63–65]. We acknowledge the importance of investigating the outcomes of other NCD in the context of the COVID-19 pandemic.

## Conclusions

The findings of the study indicate that excess mortality from all causes was significantly higher than COVID-19-related deaths reported in Latvia during the first year of the pandemic. Several factors, including insufficient testing capacity, substantial reductions in healthcare services, delayed care, and misclassification of COVID-19 deaths, may contribute to the underestimation of the pandemic's total burden.

This is the first study on excess mortality from noncommunicable diseases during the first year of the COVID-19 pandemic in Latvia. Estimations of excess mortality from NCD, particularly circulatory disorders, suggest an adverse effect of the COVID-19 pandemic on NCD patients during the pandemic's first year. Significant excess mortality from circulatory diseases might result from a combined effect of COVID-19 exposure and reduced access to healthcare services for NCD patients. Subsequent investigations of the long-term effects of the COVID-19 pandemic on NCD patients are necessary. In addition, an in-depth analysis of cause-specific excess mortality for different NCD and population subgroups is important to raise awareness of the impact of COVID-19 on public health and inform policy.

Monitoring excess mortality increases understanding of the pandemic's effect on public health. For this purpose, public health and healthcare data systems and research should be strengthened and supported to provide timely and high-quality evidence-based information necessary for effectively responding to and addressing public health emergencies.

## Supplementary Information

**Additional file 1.** 

**Additional file 2.**

## Data Availability

The dataset used and analysed for the current study is available from the Centre of Disease Prevention and Control (CDPC) of Latvia, and restrictions apply to the availability of these data, which were used under licence for the current study and are not publicly available. The datasets of aggregated data used during the current study are available from the corresponding author upon reasonable request after receiving permission from the CDPC Latvia. The R scripts of the GAM analysis used in the current study are available at the GitHub repository https://github.com/aavotins/C19_EM_LV20.

## Footnotes

1 R scripts of the GAM analysis used in the current study are available at https://github.com/aavotins/C19_EM_LV20.
