# Supplementary material for: Excess mortality associated with the COVID-19 pandemic in Latvia: a population-level analysis of all-cause and noncommunicable disease deaths in 2020
Source: BMC Public Health. 2022 Jun 3;22:1109. doi: 10.1186/s12889-022-13491-4 (PMC9163859; doi:10.1186/s12889-022-13491-4)
Supplement: Supplementary file 1 — Additional file 1. [file 12889_2022_13491_MOESM1_ESM.docx]

**Additional file 1**

Table 1 – Additional file 1. Descriptions of the best GAMs of expected all-cause and cause-specific mortality from selected noncommunicable diseases in Latvia from 2015 to 2020^a^

|  |  | All-cause | Circulatory diseases | Malignant neoplasms | Diabetes mellitus | Chronic lower respiratory diseases |
| --- | --- | --- | --- | --- | --- | --- |
| Year | 2015 | Reference | reference | reference | reference | reference |
|  | 2016 | 1.01  (0.99–1.03) | 0.99  (0.97–1.01) | 1.01  (0.98–1.05) | 1.17  (1.04–1.33) | 1.21  (1.03–1.41) |
|  | 2017 | 1.03  (1.01–1.05) | 0.98  (0.96–1.00) | 1.04  (1.00–1.08) | 1.24  (1.10–1.41) | 1.19  (1.02–1.39) |
|  | 2018 | 1.04  (1.02–1.06) | 0.98  (0.96–1.01) | 1.04  (1.00–1.08) | 1.27  (1.13–1.44) | 1.21  (1.03–1.41) |
|  | 2019 | 1.01  (0.99–1.03) | 0.93  (0.91–0.96) | 1.04  (1.01–1.08) | 1.42  (1.26–1.60) | 1.02  (0.86–1.20) |
|  | 2020 | 0.94  (0.91–0.96) | 0.88  (0.85–0.91) | 1.03  (0.97–1.09) | 1.29  (1.07–1.56) | 0.93  (0.72–1.20) |
| WOY | basis function | tp^b^ | tp | cc^c^ | cc | tp |
|  | edf ^d^ | 11.96 | 11.17 | 2.37 | 2.15 | 4.60 |
|  | ref. df^e^ | 14.94 | 13.95 | 50 | 50 | 5.75 |
|  | Chi-squared | 905.8 | 775.3 | 11.72 | 8.66 | 52.03 |
|  | p value | <0.001 | <0.001 | 0.001 | 0.006 | <0.001 |
| R-sq. (adj.)^f^ | | 0.65 | 0.67 | 0.04 | 0.11 | 0.16 |
| Deviance explained (%) | | 67.2 | 68.0 | 8.53 | 15.4 | 18.2 |

^a^ GAM best model results on mortality relative risks with 95% confidence intervals

^b^ tp – thin-plate splines

^c^ cc – cyclic cubic splines

^d^ edf – effective degrees of freedom

^e^ ref. df. – reference degrees of freedom

^f^ R-sq. (adj.) – adjusted coefficient of determination
