# Supplementary material for: Excess mortality associated with the COVID-19 pandemic in Latvia: a population-level analysis of all-cause and noncommunicable disease deaths in 2020
Source: BMC Public Health. 2022 Jun 3;22:1109. doi: 10.1186/s12889-022-13491-4 (PMC9163859; doi:10.1186/s12889-022-13491-4)
Supplement: Supplementary file 2 — Additional file 2. [file 12889_2022_13491_MOESM2_ESM.docx]

**Additional file 2**


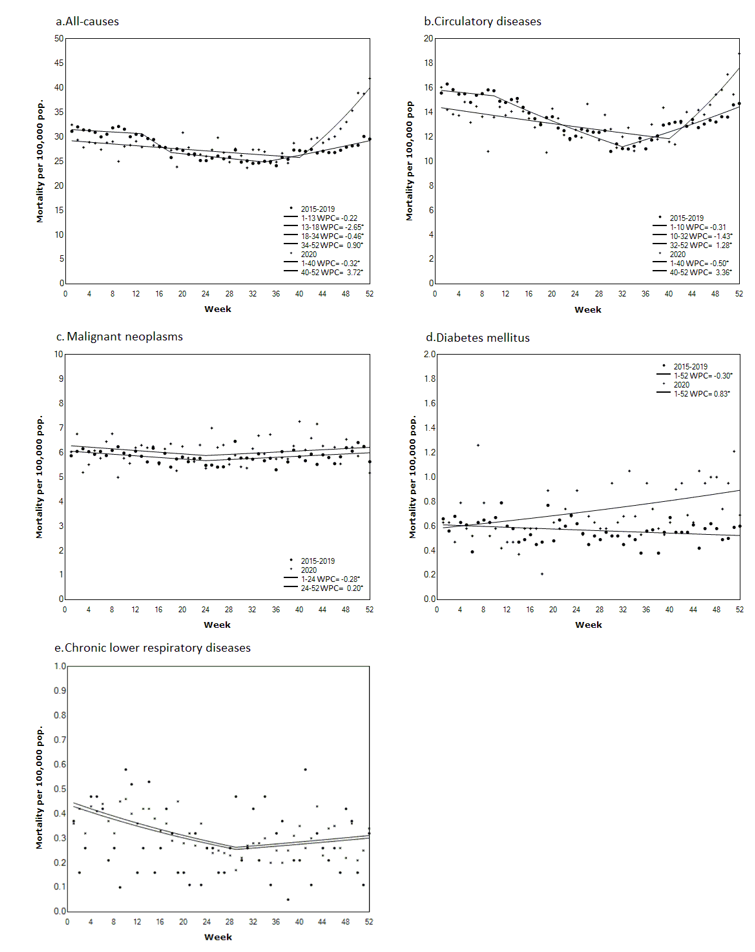


Figure 1 – Additional file 2. Mortality trends in Latvia in 2020 compared to the average from 2015 to 2019: Joinpoint regression analysis

a. All-cause

b. Circulatory diseases

c. Malignant neoplasms

d. Diabetes mellitus

e. Chronic lower respiratory diseases
